# “Whatever is in the ARVs, is Also in the PrEP” Challenges Associated With Oral Pre-exposure Prophylaxis Use Among Female Sex Workers in South Africa

**DOI:** 10.3389/fpubh.2022.691729

**Published:** 2022-06-17

**Authors:** Nosipho Faith Makhakhe, Yvonne Sliep, Anna Meyer-Weitz

**Affiliations:** ^1^Faculty of Arts and Design, Center for General Education, Durban University of Technology, Durban, South Africa; ^2^Health Economics and HIV/AIDS Research Division (HEARD), University of KwaZulu-Natal, Durban, South Africa; ^3^Department of Psychology, School of Applied Human Sciences, Howard College, University of KwaZulu-Natal, Durban, South Africa

**Keywords:** female sex workers, HIV, pre-exposure prophylaxis (PrEP), ARVs, stigma

## Abstract

**Background:**

The national policy on oral pre-exposure prophylaxis (PrEP) for female sex workers (FSWs) was instituted in South Africa in 2016. FSWs were targeted for PrEP due to a Human immunodeficiency virus (HIV) prevalence of 57.7%, which is higher than the prevalence of 19.07% among the general population. Research from demonstration studies has shown that uptake of PrEP has been slower than anticipated, and the purpose of this study was to explore barriers to the uptake of PrEP among FSWs.

**Methods:**

An in-depth qualitative study was conducted with 39 participants, 30 individual participants, and nine focus group participants. Eleven participants consisted of peer educators and two health workers from a sex work and PrEP distribution organization, the rest of the participants (1) were FSWs.

**Results:**

The majority of participants mentioned that little distinction was made between PrEP and antiretrovirals (ARVs) taken by FSWs living with HIV. PrEP was not distributed through public health care facilities, and health workers unknowingly labeled PrEP as ARVs. Consequently, health workers seen as experts created suspicion and contributed to the mistrust of PrEP among FSWs due to mixed messages, and equating PrEP to ARVs reduced uptake. Furthermore, failure to make a clear distinction between oral PrEP and ARVs resulted in accusations of lying, denialism, and deception leveled at users of PrEP by FSWs using ARVs, and sometimes by clients and intimate partners. FSWs using PrEP reported feeling stigmatized and thrust into interpersonal conflict with their peers after choosing PrEP, leading to broken relationships, and some resorting to taking PrEP privately or discontinuing PrEP altogether.

**Conclusion:**

Pre-exposure prophylaxis as an ARV targeted for the prevention of HIV among high-risk groups was found to be stigmatizing. The distinctive use of PrEP and ARVs should be correctly explained to users to minimize confusion, enable differentiation and reduce interpersonal conflict. Cohesion among sex work organizations and public health care facilities is needed to disseminate the correct knowledge on PrEP. A non-stigmatizing approach to the distribution of PrEP may serve to increase uptake and adherence.

## Introduction

Globally, the goal is to end AIDS by 2030. Significant declines in AIDS-related deaths have been observed among people from all ages living with HIV, from a staggering 1.9 million in 2004 to 940,000 in 2017 (2). The decline in AIDS-related deaths is mainly driven by the upscaling of ARV therapy, which is currently being made available immediately to those who test positive (test and treat) to achieve viral suppression and curb transmission (2). A significant decline of global deaths from AIDS-related illnesses has been observed, particularly in sub-Saharan Africa, and more specifically in Eastern and Southern Africa, which is considered as the epicenter of the epidemic. It is said to have 53% of the world's population living with HIV, with the epidemic being higher among women (56%). AIDS-related mortality declined by 42% from 2010 to 2017 due to the increase in treatment in this region (2).

Despite the progress made in the decline of AIDS-related deaths and ways to limit new infections, there are still populations among which the epidemic is still highly concentrated. These are key populations which require strategic focus (3). The added complexity with these populations is that they are hidden because their lifestyles are either criminalized or deemed morally unacceptable, which makes it challenging to design prevention strategies for these groups (3). Data has shown that 47% of new HIV infections worldwide are associated with key populations and their sexual partners (2). Research, specifically among FSWs in sub-Saharan Africa, has shown that approximately 37% of FSWs are HIV positive due to exposure to a number of multiple intersecting vulnerabilities such as multiple sexual partners, lack of access to health care because of stigma and discrimination, violence from partners and clients, as well as consumption of drugs and alcohol as a coping mechanism (3).

In South Africa, HIV prevalence among FSWs is said to be at 57.7%, which is higher than the estimated national prevalence of 19.07% (2, 4). Thus, the South African government, with the aid of the World Health Organization guidelines, instituted the distribution of pre-exposure prophylaxis (PrEP) among FSWs (5). There is substantial research that has investigated the barriers and facilitators for uptake, and adherence to retention of PrEP among men who have sex with men (MSM) (6–11). Various studies from different contexts have documented challenges to PrEP adherence and retention among minority groups, adolescents and serodiscordant couples (12–14). However, only a few studies from the South African context have looked at barriers and facilitators of PrEP use among FSWs. These are studies among FSWs in one part of the country (Gauteng province) (15–17). Thus, more studies are needed to understand the challenges to PrEP use among FSWs in other parts of South Africa. This study was conducted in Durban KwaZulu-Natal (KZN), the mapping of epidemiological data in the UNAIDS data report (2) has revealed that KZN has a high distribution of HIV infections, making it a hotspot where 40.8% of people aged 15 years and older are living with HIV. Furthermore, the mapping showed that people within this geographical area have a 46% higher risk of acquiring HIV than people living outside of this region (2). A respondent-driven sampling revealed a 53.7% HIV prevalence among FSWs in Durban (18). This is an indication of the importance of this PrEP study conducted among FSWs in this region. Furthermore, in this region.

Pre-exposure prophylaxis knowledge among FSWs is sparse and distribution is limited, therefore this study provides insight into the challenges that FSWs experience with taking PrEP as a form of HIV prevention. Data from this study can help inform the design and implementation of PrEP interventions among FSWs.

## Methods

### Study Setting and Background

This study was conducted in the coastal city of Durban in KwaZulu-Natal (KZN). KZN is one of the nine provinces in South Africa, and Durban is the third largest city in South Africa with over 3,442,398 people. It has one of Africa's busiest harbors and is a major tourist destination with its warm, subtropical climate and extensive beaches (19).

This formative study was conducted from May to November 2018, with the purpose of understanding FSWs' experiences with PrEP. It also sought to capture the experiences of PrEP service providers with regard to how service users were responding to PrEP as an additional HIV prevention method. This study was conducted among women who sell sex in the city of Durban. The FSWs who participated in this study worked mainly in indoor establishments such as hotels and private houses, with a few women working in both indoor establishments and on the streets. The researcher as well as the counselor and peer educators interviewed were from two organizations: *Sisonke* and *TB HIV Care*. Both these organizations serve the health care needs of FSWs. *TB HIV Care* promotes and distributes PrEP to FSWs in this region through peer education.

### Research Design

A qualitative study was conducted based on the social constructionist approach. Qualitative research aims to examine and understand people's experiences and perceptions about a specific phenomenon in the social world. One of the distinct features of qualitative research is that it enables the researcher to explore issues from the perspective of the study participants; hence, the social constructionist approach as it centers participants as experts in their own lived experiences. In order to develop a deeper understanding of a particular social issue, the researcher relied on the understanding, meanings, and interpretations of study participants regarding a particular behavior, event, or object (20–23).

### Sampling Strategy

Snowball sampling was the primary sampling method used to locate participants. Snowball sampling is a non-probability type of sampling that is used among hidden populations (24); in this case FSWs. It is a chain-referral method where one participant refers others into the study. This chain referral begins with a purposive sample of initial subjects who then refer other members of the population to the study (24, 25). This sampling method is often used for groups that are stigmatized and involved in criminalized or deviant behaviors (22, 26). The limitation with this sampling method is that it makes it difficult to break into other networks because recruitment takes place in one network of friends or acquaintances (1). The study, however, relied on two groups of peer educators from two sex work organizations *Sisonke* and *TB/HIV Care*, who had access to various networks of FSWs because of their outreach work, and it was anticipated that drawing from these networks would bring in FSWs from indoor and outdoor establishments with varying experiences.

In order to gain entry into the target population and to start the snowball process, one peer educator from *Sisonke* was purposively sampled. Purposive sampling is defined as a deliberate process of selecting individuals or groups that are knowledgeable and well informed about the phenomenon under study (27, 28). After interviewing this peer educator, other peer educators from *Sisonke* snowballed into the study. The participants were interviewed based on their willingness to participate and their ability to communicate experiences and opinions in a detailed and articulate manner to provide rich and valuable data to gain a deeper understanding about PrEP use among the sex work population (29).

The peer educator from Sisonke that was purposively sampled, introduced the researcher to the *TB HIV Care* site coordinator in charge of the PrEP program who agreed to participate in the study. Through the site coordinator, the researcher was put in contact with the rest of the peer educators and a counselor from within the same organization who were willing to participate in the study. The other FSWs who participated in the study were linked *via* other networks of peer educators from both *Sisonke* and *TB HIV Care*.

### Sampling Description

The study sample consisted of a total of 39 participants. This sample was determined through data saturation. Data saturation is reached when there is enough data to replicate the study as no new information emerges from the interviews conducted, and extended coding is no longer feasible because there are no new emerging themes that arise from the interview data (30, 31). In order to reach data saturation, it is important to pose the same key interview questions to multiple participants. It is also important to elicit information from other data collection methods, in this case focus group discussions and key informant interviews to get multiple perspectives about a phenomenon, as well as collecting data from different stakeholders. This assisted the researcher in concluding whether a consistent message is communicated by the respective participants (32).

There was a total of 30 in-depth key informant interviews with 11 peer educators, one site coordinator and one counselor (thus, a total of 13), while the rest were FSWs (18). Nine other FSWs formed part of the focus group discussions (Group 1, five members and Group 2, four members). In total, 26 FSWs were interviewed, of which 20 were on PrEP, with two having defaulted but had resumed with PrEP at the time of this study. Another two who were using PrEP were dependent on drugs and were sober at the time of participating in the interviews. Six FSW participants were not on PrEP.

### Data Collection Tool, Processes, and Procedures

One-on-one in-depth interviews and focus group discussions were conducted in English and isiZulu. A semi-structured interview guide was used as a data collection tool for both in-depth interviews and focus group discussions. Using semi-structured interviews is a popular data collection method because it is versatile and flexible and can be utilized for individual and group discussions (33). The semi-structured interview format allows the researcher to probe so as to explore an issue with greater detail and this is done through asking follow-up questions and allows the participants to express themselves freely (34, 35). Through the questions posed from the semi-structured interview guide, it was possible to elicit the sociodemographic information of participants and understand the process of PrEP outreach, education, initiation, and efforts to encourage retention among FSWs. Through the interviews and focus group discussions, a deeper understanding of the environmental, personal and interpersonal challenges in the uptake of PrEP among FSWs could be obtained. Individual interviews were approximately 40 min long and focus group discussion were about 60 min long.

The scheduling of interviews was through two specific peer educators from *Sisonke* and *TB HIV Care*. Telephonic contact to prospective participants was made by a peer educator. The peers explained the aims and objectives of the study to the participants and to ascertain their willingness to participate in the study. Arrangements were made for the physical location where the interviews and group discussions were to take place where the researcher was introduced to participants prior to the interviews. Some interviews and focus group discussions took place at both the *TB HIV Care* and *Sisonke* offices. The participants felt comfortable coming to these offices because of their safe and familiar environment. Participants were used to coming to *TB HIV Care* for medical care and counseling and some accessed Sisonke offices to report human rights violations. They also came to attend psychosocial creative space meetings. Both offices provided a private room for the interviews. Some participants who could not come to the *TB HIV Care* offices were interviewed at their homes and others were interviewed at the brothels where they worked. One of the peer educators was generous enough to offer her time and accompany the researcher to see these participants. She was a valuable guide because some of the areas were unsafe.

Consent forms were handed out to participants prior to each interview and focus group discussion. The contents were explained as some participants could not read. Consent forms were translated into isiZulu because it is the main language spoken in Durban. Participants were made aware that their identities would be kept confidential and that their participation was voluntary; they were free to stop participating at any point during the interview. The participants were also made aware that they would be reimbursed for their time. It was mandatory for a participant to sign both the consent form for participating and another form agreeing to the audio recording of the interview or discussion. Most participants accepted to be recorded, except for one participant who was not comfortable and therefore notes were taken during that interview. The participants were also made aware that their recorded interviews would be stored electronically on an external hard drive which will be kept in a secure office along with the transcript for a period of 5 years, and that only the researcher and supervisor would have access to the files. Ethical clearance for this study was obtained from the Humanities and Social Sciences Research Ethics Committee at the University of KwaZulu-Natal (IRB number HSS/0203/018D).

### Data Analysis

All audiotaped data were transcribed and translated verbatim by a research assistant with an honors degree in psychology who was proficient in both English and isiZulu. The transcripts were generated after each interview. Quality checks were done as well as to identify patterns or inconsistencies in the data, which were addressed with the next group of participants.

The data was analyzed thematically. This involved sorting and coding the data into themes and categories by identifying and analyzing repeating patterns that existed in the data (36). Themes are important patterns emerging from the data that are related to the research objectives and help to answer the appropriate research questions (36). The analysis followed six steps: to translate and transcribe the data verbatim, familiarizing oneself with the data by reading and rereading the data so as to generate codes, generate themes from the codes, define and refine the identified themes, and employ the identified themes in the final presentation of the study findings (36). Transcripts were read and codes were generated from each transcript, all codes were listed, and similar codes were clustered together and constructed into themes and subthemes. Themes and subthemes were entered into an excel spreadsheet. Each Excel spreadsheet consisted of a particular theme and related subthemes. One Excel spreadsheet consisted of themes and subthemes from interviews with peer educators, researcher and counselor and another spreadsheet consisted of themes and subthemes from interviews and focus group discussions with FSWs. These spreadsheets were populated with various quotes related to the specific themes and subthemes as communicated by the participants. Each quote was marked with an interview number and each participant was given a unique identifier. This helped in discerning what the various participants were saying concerning a particular theme. One researcher conducted the data analysis process guided by two PhD advisors. Discussions were held between the researcher and advisors pertaining to the transcribed data as well as the codes that were identified from the data and the themes that were developed and presented in this paper.

### Data Verification Process

The process of data verification included triangulation which encompasses the use of two or more data collection methods. For the purposes of this study the researcher compared and contrasted data from the focus group discussions as well as data from the in-depth interviews. This helped to compensate for various limitations encountered with each data collection technique. The researcher also conducted member checks in the form of a data verification meeting with FSW peer educators who participated in this study, unfortunately not all participants in the study were able to attend the data verification meeting. The themes and subthemes which included participant quotations were presented and each theme was discussed with the group to verify whether the researcher had the correct interpretation of the data. This process was important as it provided the researcher with an opportunity to address any preconceived biases that may have clouded the researcher's judgement during the data analysis process.

### Sociodemographic Background of Participants

Table 1 shows the sociodemographic background of the participants.

**Table 1:** Sociodemographic background of participants.

**Participants**	**Female sex**	**Female sex**	**Health care**
	**workers**	**worker peer**	**providers/**
		**educators**	**researcher**
**Gender**			
Female	26	11	1
Male	0	0	1
**Age groups**			
19–23	1	0	0
24–28	6	3	0
29–33	4	0	0
34–38	11	2	2
39+	4	6	0
**Educational level**			
Primary school	0	1	0
Secondary school	16	5	0
Matric (Grade 12)	10	4	0
Tertiary	0	1	2
**Home language**			
isiZulu	17	8	1
isiXhosa	2	2	0
Sesotho	1	1	0
English	1	0	0
Other^*^	5	0	1
**Racial group**			
Black	25	11	2
Mixed race	1	0	0
Indian	0	0	0
White	0	0	0
**Country of origin**			
South Africa	21	11	1
Zimbabwe	5	0	0
Congo	0	0	1
**PrEP uptake**			
Taking PrEP	20	1	0
Not taking PrEP	6	10	2
**Total number of participants:** 39			

* *Foreign nationals*.

## Results

**Figure 1:**
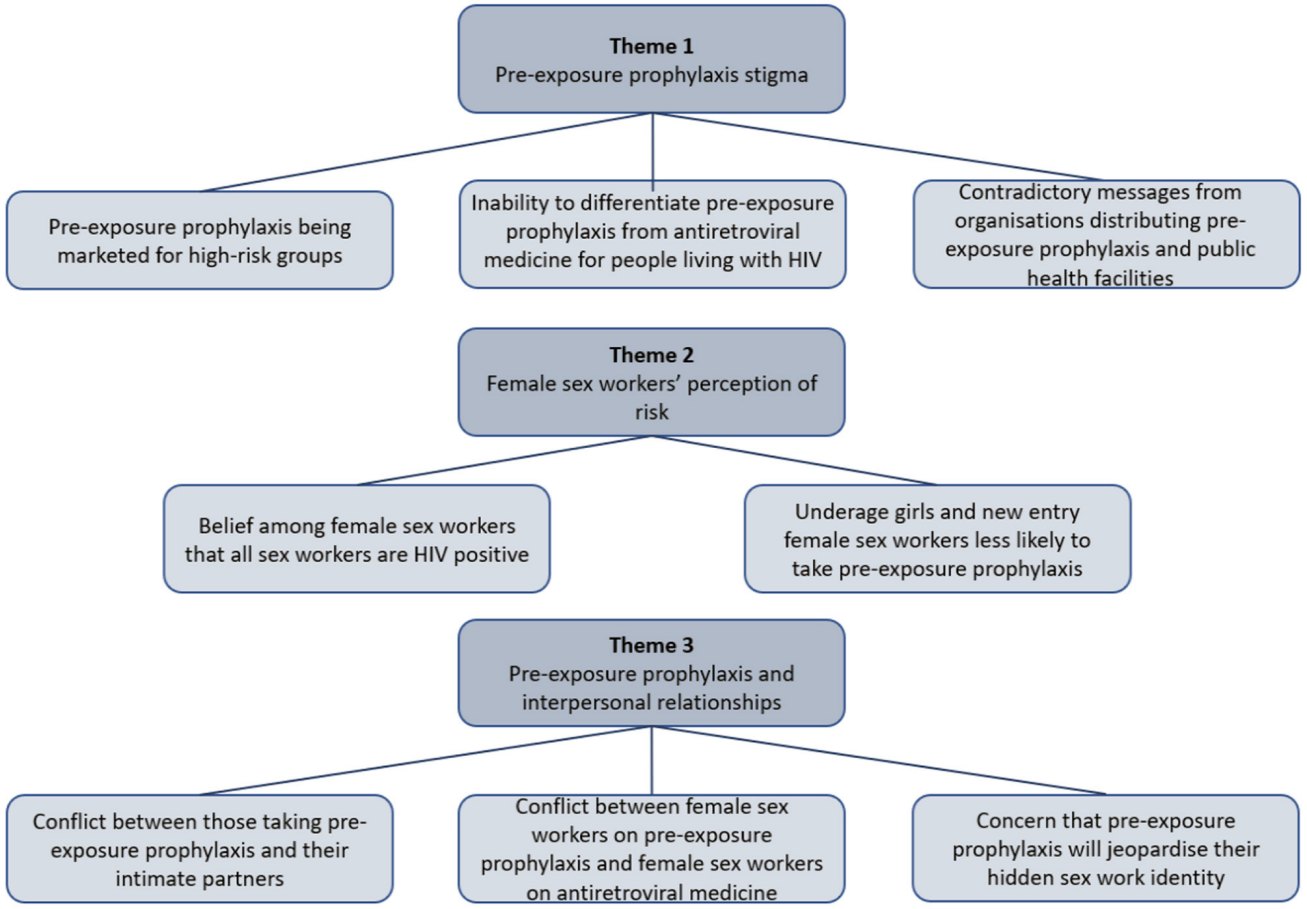
Themes and subthemes.

The results for this study are discussed under the following themes as depicted in Figure 1.

### Pre-exposure Prophylaxis Stigma

The subthemes identified in the data pertaining to stigma were PrEP being marketed for high-risk groups, inability to differentiate PrEP from ARVs for people living with HIV, as well as contradictory messages from organizations distributing PrEP and public health facilities.

#### Pre-exposure Prophylaxis Being Marketed for High-Risk Groups

With the exception of one person, all the participants agreed that PrEP being marketed and distributed specifically to high-risk groups is stigmatizing. The participants felt that the emphasis on high-risk groups reinforced the belief that FSWs are the carriers of HIV:

I can really say that stigma on HIV is still prevalent in our society. Now, for any person taking PrEP, when PrEP is not well known, this is difficult. The fact that PrEP is not well known to the general population creates a big stigma and saying that you are taking ARVs, this may be difficult to explain to any person (Researcher age 35)

I think once they take off that thing, that this thing [PrEP] is for people who are really at risk which categorizes sex workers only, it will be fine with everybody because they will start giving education like even on the radio, like they are doing with ARVs … (Counselor, age 37).

I think you can say this pill is for people who are sexually active, it does not matter how old you are, you are young, old, a sex worker, but as long as you partake in sexual activities you must know that you must find a way to protect yourself (FSW peer educator, age 50).

Furthermore, the participants argued that a wider distribution of PrEP would lead to the normalization of PrEP as an HIV prevention option and this may help to curb stigma, and increase the likelihood of PrEP uptake among FSWs:

The government should give it to everyone just like they do with the ARVs, and it must not be specifically given to sex workers … (FSW peer educator, age 42).

They can advertise PrEP everywhere. Right now, there is nothing-nothing that's being done. We as mobile clinics, we as organizations, we need to push, we need to go out and tell people about PrEP (Counselor, age 37).

Participants strongly felt that there are other women in need of PrEP but are being overlooked due to the focus on FSWs. Those who engage in transactional sex with multiple partners, those at risk but do not identify themselves as sex workers, as well as married women who may not be able to negotiate condom use with husbands that engage in multiple sexual relationships:

We have got various sex workers, some do it in the clubs, some do it in the neighboring areas in a community and we have ‘sex workers’ who have never come out in public/admitted that they are sex workers. She does not know she is doing ‘sex work’, why, because she has never carried her handbag and went out to sell sex and that person is at high risk because she is the one who does not wear a condom totally because she is deceiving herself. She's scared to make money publicly the way I am making it (FSW peer educator, age 42).

Yes, I think it is because there are married women out there who need such protection [PrEP]. I was once married before and got divorced, both my kids are from wedlock. So, if you are married you cannot say no to unprotected sex, so married women can also get infected by HIV, because some men are not faithful. So, these pills must be given even in public health sectors, to avail it to those who are faithful and want to protect themselves, especially married people. I feel sorry for them because they have got no say when it comes to sex, their men want to have unprotected sex with them while they know that they sleep around with multiple partners… (FSW, age 38).

The wider distribution of PrEP was viewed as a solution to increase confidence in PrEP efficacy and lessen the current suspicion that PrEP is a drug being tested on FSWs simply for scientific purposes:

Now as a scientific organization the suspicion is that we are simply testing PrEP on FSWs and this makes it difficult for them to accept it (Researcher, age 35).

#### Inability to Differentiate Pre-exposure Prophylaxis From Antiretroviral Treatment for People Living With HIV

Due to PrEP being essentially an ARV pill (tenofovir), the participants felt that this contributed to further confusion among FSWs and led to the difficulty in PrEP uptake and adherence, because some sex workers could not tell the difference between the two regimens (PrEP for prevention and ARVs for people living with HIV). This compounded the fear of stigma and cast a sense of doubt among those who were keen to take up PrEP because of its association with ARVs, which are stigmatized:

Some are understanding, and some are still not understanding, we tell them but still they have not understood it. They have not understood it because we give others PrEP and when they open it, others will come and tell them that it's ARVs. We tell them that these are not ARVs because the color is not the same and PrEP has two components because it is for protection, and the ARVs has three components for treatment. That is how we tell them and some default because they think it's ARVs…. (FSW peer educator, age 46).

They [FSWs] are also saying that whatever is in the ARVs is also in the PrEP, so they are the same (FSWs, age 26).

PrEP prophylaxis as tenofovir, which is an ARV, has created a situation where some FSWs felt that there is no difference between someone who is on ARVs and someone who is on PrEP because the adherence requirements for both mean a pill a day at a specific time. Thus, an FSW on PrEP also engages in a similar daily routine as an FSW on ARVs. This reinforced the idea that the two regimens are the same, as expressed by the following participants:

The stigma is around the fact that tenofovir [PrEP] is an ARV, when a person actually googles it, it is going to give all the options of an ARV, now the question out there we can say it's 2018, everyone knows about HIV but trust me, still few people are accepting the fact (Researcher, age 35).

Me, one day I didn't want to take the tablet because the person who takes an HIV tablet and the person who takes PrEP are not different. Why I say so is because when I compare those tablets, I have one of my friends who is taking the HIV [ARV] tablets. When I compare there is no difference. They look the same, the size, the way they are, everything. So, one of my friends asked me “Why are you taking those pills because you are not HIV positive?” I said I just want to protect myself. She told me: “Which means we are the same. Why are you still taking meds?” I said it's for preventing HIV. Then they went on Google. They googled that tablet and there was so much information. You know people can discourage you saying these pills are no different from the ones for HIV [ARVs] there is only one difference, but all these things are the same…(FSW, age 33).

At times you end up explaining but some will not understand and ask why I am taking pills that they have never seen in public clinics before, and I keep on saying I am not sick. Can you imagine that thing? A friend of mine is also teasing me that I should just start eating ARVs because this thing is the same as ARVs. I think if it can be provided in public clinics then it will be okay. It would be good (FSW, age 35).

As expressed by participants, PrEP needs to be made available to anyone who is at risk for HIV and not only among specific groups. This will prevent the association of PrEP with sex work or with being HIV positive. It is also important for PrEP education to be explicit about PrEP being a tenofovir, which is an ARV regimen that can be utilized for HIV prevention.

#### Contradictory Messages From Organizations Distributing Pre-exposure Prophylaxis and Public Health Facilities

Another challenge that contributed to the stigmatization of PrEP among FSWs was that most health care professionals that participants came across in public clinics and hospitals, did not know about PrEP, and simply labeled it as an ARV, which discouraged FSWs from taking it. In addition, the fact that PrEP was being distributed through one local non-governmental health research organization that is known to target FSWs with health care services, created a situation where FSWs were curious as to why a vital pill that is said to prevent HIV was not being widely distributed to the general public through government health facilities. This created another layer of confusion among FSWs who went to their local clinics to enquire about PrEP for more information to gain a sense of clarity. These factors contributed to skepticism among FSWs regarding PrEP:

Even at public clinics, sometimes the nurses don't know what PrEP is, because the other lady said the nurses said this is an ARV, yes, there are two combinations of the ARVs in PrEP, but it is not an ARV for positive clients, that's the problem, now the nurses also discourage them in the public clinics and tell them that this thing is an ARV. The FSW will phone me back and say: “I'm not taking this thing because even the sister [nurse] told me that this thing is an ARV… I think even in the hospitals they need something like a workshop, they need to be taught about PrEP…(PrEP counselor, age 37).

Nurses at public clinics do not know about PrEP and even people from townships do not know, because I have also experienced some challenges with my neighbors. The fact that you know that they [PrEP] are not HIV treatment you will just place them anywhere in the house, not that you are proud of being negative, but when your neighbor comes in and see it, she will just ask if you are also taking ARVs, some will say you have finally found this sickness, and you do not know how to explain to them because they are clueless (FSW, age 34).

Furthermore, the contradicting messages from public health care providers and the PrEP distributing organization contributed to lower retention rates because the FSWs who were initially enthusiastic to take up PrEP, experienced discouragement from the public health care professionals who simply dismissed PrEP as an ARV, without explaining that PrEP is for HIV prevention because of their inadequate PrEP knowledge. The lack of coherence that existed between the two health care structures resulted in unintended consequences that slowed down PrEP uptake and retention among FSWs:

We had issues with people accepting PrEP because somebody will say it is only for sex workers and until now there's another organization that is also in eThekwini [Durban] that has started to implement PrEP among students, for example. However, all this time it was not done, meaning it was only sex workers who were actually getting PrEP. So, now because there was no sensitization done to our local clinics, and the lack of information from our health professionals, the health professional is not just a nurse. I'm referring to the level of doctors, clinicians basically, if they do not understand what it is, they will just tell you to stop that treatment, these NGOs [non-government organizations] are giving you, just stop it. This ended up being one of the reasons that our retention of PrEP dropped drastically… (Researcher, age 35).

### Female Sex Workers' Perception of Risk

The themes that will be discussed under risk perception are the belief among female sex workers that all sex workers are HIV positive, as well as limited risk perception among underage girls who enter sex work and new entry sex workers being unlikely to take PrEP.

#### Belief Among Female Sex Workers That All Sex Workers Are HIV Positive

The majority of the participants mentioned that there is a general misconception among FSWs that everyone who is a sex worker is HIV positive; therefore, as mentioned above, being in possession of PrEP is associated with being HIV positive. First, this belief resulted from the fact that these women shared clients, and condom use was inconsistent:

Most girls like deceiving themselves and say we are all sick and if you tell them that you are taking PrEP, they will make you a laughingstock and say you are fooling yourself by taking PrEP, thinking that you are HIV negative. Some understand, and some do not, and they also laughed at me that I am fooling myself, why am I not taking ARVs. So, the belief is that we are all sick (FSW, age 27).

They talk because my other friend found out that she is positive, but she hasn't started taking ARVs. So, when I was experiencing rash with PrEP, she said these pills are not good. I was hoping to take them too, and I asked which pills you are going to take because these ones are meant to protect against HIV. She said no, these pills are for HIV, I have seen them from other people, and I said no they are not for HIV, and she said “no, it's because you guys are keeping it a secret and you can't accept that you are sick”, so she was quick to judge me and it is common that people will judge you and even some clients when they see you, they will just say you are taking ARVs (FSW, age 32).

Furthermore, the idea of a daily pill that is essentially an ARV that prevents HIV seemed highly improbable. The FSWs on PrEP were accused of HIV denialism or having blood type O, which among this population is understood as a blood group that cannot be infected with HIV. The group mentality that all FSWs are HIV positive could lead to a misguided perception of risk or apathy and FSWs may therefore not take adequate precautions to prevent HIV:

There is a girl I shared a boyfriend with. I found them together, the girl was taking ARV pills. Therefore, when I told her I was on PrEP, she asked how come? And I explained, she then said maybe I am blood type O and whatnot. I told her there is no such thing about blood types or whatever, and nobody believed me when I told them I don't have the virus (FSW, age 27).

Participants who were on PrEP mentioned that they were surprised that they tested negative because of the risks incurred in sex work. Others believed that the HIV was hiding in their blood and it was only a matter of time until it showed itself. Thus, those taking PrEP were told by their friends to stop wasting their time and start taking ARVs since it was highly possible that they were HIV positive:

Maybe it [HIV] could be somehow hidden in my system, but when the sisters [nurses] who usually come here paid us a visit I asked if it is possible for the virus to not be detected in my system. They said it is rare, especially if your blood type is susceptible to it. So, before I started taking PrEP, they also run me some blood tests and they said there is no such, if you are positive or negative nothing will change that … (FSW, age 27).

They will say that you think you are better. That is what my friend told me, she said: “It's not like you are HIV negative, but it's because it is still invisible in your system” (FSW, age 35).

In the face of misconceptions, there were participants who refuted the myths and felt strongly about taking PrEP. They trusted that they were HIV negative and focused on protecting themselves by taking PrEP consistently:

So, there is a lot of peer pressure to say all of us are positive even though it's not true, but the belief that “we are all doing the same job then why are you negative and why am I positive?” It's like there is that thing that we are all the same. But they used to tell me: “You are taking these tablets and still working in this business but one day you will get it.” I just tell myself that I will not get it because I know what I am doing… I have to keep taking those PrEP tablets. I can't stop them (FSW, age 33).

I just have that attitude that I know myself and I don't need someone to tell me what to do. Yes, it is true that if you take bottles for both PrEP and ARVs, they are almost in the same bottles, so they say why are they giving you this pill if you do not have the virus? So, it's either you got it too, or it is invisible, or they are giving you pills to hide HIV and it will come out later. So, it is better to take ARVs because you know that you are taking ARVs, then to think you are clean when you know that you are sick. It seems like there is a feeling that because we are doing the same job, we must all be infected (FSW, age 34).

### Underage Girls and New Entry Female Sex Workers Less Likely to Take Pre-exposure Prophylaxis

There were participants who were adamant that young girls who sell sex and new entry FSWs were less likely to take PrEP because of their low perception of HIV risk and thus they do not engage in preventative behaviors as they might be oblivious to the dangers involved:

They only see later after experiencing some challenges, but when they are new, especially the young girls who are entering the industry they are not aware of the risks in this job. What happens is that you may find that these girls are young … (FSW, age 26).

The young, underage girls who sell sex were viewed to be financially desperate and eager to make money, even at the expense of their own health. Their risk is compounded by high levels of client demand, and because of being new to the trade they are considered “fresh” and therefore more desirable to clients. Their inexperience with negotiating condom use with clients also puts new recruits at a disadvantage and a likelihood of contracting HIV:

Clients are after those who are new since they are fresh, and they do not have an experience with this job [selling sex]. Clients do not want people like us who are old and have a lot of experience, so these young girls only realize the risk of doing this job when they are being infected by sexually transmitted infections and other diseases, that is when they discover that this job is risky, but as long as they are getting money to buy whatever they need, they will not even listen to us even if we are advising them (FSW, age 26).

Women who are new to the sex trade, were also categorized by participants as high risk for HIV and less likely to take precaution because of being in denial about doing sex work, they do not immediately acknowledge their multiple sexual interactions as sex work:

Firstly, when you are new in this industry you do not even tell yourself that you are doing that thing [sex work], you just take it lightly, thinking that you were just being naughty because you are desperate. Yes, you do not think about what you must do or not do to protect yourself. Also, it is not easy for a person to just go and stand on the streets or go in a certain brothel to work (FSW peer educator, age 51).

The refusal to accept that one is a sex worker means that their focus is purely on making money and not necessarily on taking the needed precautions to ensure that they remain HIV negative:

Your spirit is always pleased by the money you are receiving/making that you have never had before. Or maybe you have been doing this job, but you did not know you were doing it because it happens that you have five boyfriends, and this is part of this job because among those five boyfriends, it is not like you love them all, among those five boyfriends, one of them might not want to use a condom, maybe three or two of them can ask to use a condom. So, you may not realize that you might be in trouble because you are used to having sex with multiple people maybe a day, without knowing that you are being a sex worker (FSW peer educator, age 42).

In some instances, new entrants into the trade may not know of the mobile HIV prevention services available to them and may miss out on the educational prevention programs pertaining to PrEP. At times, the older FSWs would have already discouraged the new recruit from initiating PrEP because of the stigma surrounding PrEP:

The problem is that each and every year, or I can say each and every month, there is a new sex worker joining all those old sex workers, for sure there is not enough education on PrEP, so that is our challenge because every time when we go there you will find out that sometimes they have already polluted her mind, telling her to not even try this PrEP thing…(PrEP counselor, age 37).

There were those participants who took it upon themselves to explain to newcomers the challenges that they can expect in the trade and how they can go about navigating those challenges to protect themselves; however, situations differ because the industry is unpredictable and therefore everyone has to make their own decisions based on the circumstances, they find themselves in:

We usually say to those who have been there doing it that if they bring someone here, they must tell her the challenges, tell her ways of behavior and what she must do now that she is here and what she must not do. It is easier to talk than doing it, because no matter how much I can tell you, you will follow your mind with your client because it is only you and him in the room … (FSW peer educator, age 51).

### Pre-exposure Prophylaxis and Interpersonal Relationships

The themes that are discussed under PrEP and interpersonal relationships include conflict between those taking PrEP and their intimate partners, conflict between FSWs on PrEP and FSWs on ARVs, as well as concerns that PrEP will jeopardize the women's hidden sex work identity.

#### Conflict Between Those Taking Pre-exposure Prophylaxis and Their Intimate Partners

Participants mentioned that the lack of understanding regarding the function of PrEP has led to conflict with their intimate partners with whom they have steady relationships. They asserted that when partners happened to come across the PrEP pills, their first instinct was to suspect that the female partner was taking ARVs, and they found it hard to believe that the partner was in fact engaging in HIV prevention. This misunderstanding occurred because the majority of PrEP users in this study found it difficult to speak to their partners about PrEP because they did not have the proficiency to explain its use as an ARV that prevents HIV infection:

We once separated with my partner, because he thought I was lying that this medication is to prevent HIV. I was just keeping them in a bag, and I was seeing no issue with that, and he said: “You are also taking ARVs and you do not bother telling me, because last time I checked we were both negative?” Then I told him that we must both go to the clinic and test for HIV and now he understands (FSW, age 34).

So, some of the challenges is that some ladies have boyfriends, and they will not understand if you are telling them that you are taking these pills. My boyfriend that I had said I was taking the pills because I am HIV positive, and he said he has never seen such a big pill with these colors, so I had to tell him to search and google it. Unfortunately, he was slowly coming to terms with that and we had one sister [nurse] explaining to him, because I think he took the pill and went to someone else to explain to him … But since this incident we broke up and are no longer together (FSW, age 34).

### Conflict Between Female Sex Workers on Pre-exposure Prophylaxis and Female Sex Workers on Antiretrovirals

The conflict regarding PrEP was not only experienced between FSWs and their intimate partners but it also included FSWs and their friends. This conflict was as a result of the belief that all FSWs are HIV positive as explained above. Thus, some FSWs in this study mentioned that when they opened up to their friends about taking PrEP to remain HIV negative, they received backlash and negative feedback with undertones of envy:

We are not talking to each other with my friend even now because we fought over that thing [PrEP]. She is the one who told me about PrEP, and we went for HIV testing, and when she found out that she was positive, and there was no way I could hide my status from her, so we showed each other. Then when they have started delivering our pills since they deliver where we are staying, she said it's not like you are not HIV positive it's just that … they can't find the virus in your system. She ended up hating me and saying bad things about me to other people, saying that I am taking PrEP, but I have HIV…. (FSW, age 34).

The negative reaction that FSWs taking PrEP received from their friends led some to make the decision to take PrEP secretly to avoid being ridiculed.

You end up hiding that you are taking your pills if you are still staying with people who are sex workers, because you know that you will be criticized (FSW, age 35).

#### Concern That Pre-exposure Prophylaxis Will Jeopardize Their Hidden Sex Work Identity

There were participants were genuinely concerned that taking PrEP could result in jeopardizing their confidentiality. One of the concerns was that because PrEP is for high-risk groups, it might raise suspicion among close family members if they should discover that their family member is using PrEP. Thus, some PrEP users in this study were burdened by whether to tell their families or not. This was coupled with the self-doubt of not having the ability to adequately explain the use of PrEP:

Some know about it, but a person might say: “I might take this PrEP and what am I going to say what are these drugs for because these pills are well known to be taken by only sex workers. My boyfriend or my husband does not know that I am a sex worker, so if they see me with these pills it will be clear that I am a sex worker” (FSW peer educator, age 42).

So when they are asking you where you are getting these pills [PrEP] and that you should take them to where you are getting them it's a big challenge, because you will meet people you are doing sex work with there, and some do not mind to just talk to you about sex work and you might get exposed at home just like that, and your family will know what kind of a person you are (FSW, 34).

To deal with this challenge of potential exposure, some FSWs advocated for the hiding of the pills from their families and significant others:

I have been selling sex for more than 12 years on the streets and no one has ever known that I was doing sex work. But if you are a woman, women think faster than men, so by the time I would be cooking in the kitchen, I know where I am keeping them and I know that by eight o'clock I should have eaten and have taken the pill [PrEP]. So, I will go fetch the pill where I keep it, fold it with a tissue or hide it in my apron so while I would be cooking, it would be with me, and by the time I finish eating, I will go as someone who is going to brush my teeth in the bathroom and then drink it there and I have bailed myself in that way…(FSW peer educator, age 37).

## Discussion

The participants in this study raised a number of pertinent issues that contributed to the challenges regarding PrEP uptake, adherence, and retention among FSWs. The marketing and distribution of PrEP at the time of this study was premised on targeting groups that were at high risk to HIV acquisition such as FSWs. However, the majority of participants in this study felt that this approach undermined their privacy and could potentially expose them to scrutiny from their families and partners should they decide to take PrEP. This perceived lack of privacy resulted in some FSWs feeling apprehensive about taking PrEP. Due to PrEP essentially being an ARV, this resulted into PrEP stigma, and the labeling by friends of those taking PrEP as being HIV positive. This labeling came as a result of the prevailing belief among FSWs that the majority of women selling sex are HIV positive because they share and rotate clients. This belief has resulted in FSWs questioning the efficacy of PrEP, with some going as far as not being sure whether they were truly HIV negative, given their sexual engagement with multiple partners. This doubt manifested itself through the belief and expectation that the HIV will eventually ‘show itself in their blood’. This finding was similarly reported by Eakle et al. (16). These false beliefs could lead to some FSWs accepting HIV acquisition as their fate and not acting in a decisive manner to prevent HIV infection. Furthermore, unique to this study, the participants mentioned that they were confused by the inverse notion peddled by their peers that they were probably “blood type O” and not susceptible to HIV infection. This idea is similar to findings that were released by a study published in 2015 that was conducted among FSWs in Kenya, which found that the HIV incidence was higher among blood group A as opposed to blood group AB, B and O counterparts (37). However, other studies have concluded that blood groups have no effect on HIV susceptibility or resistance (38, 39).

The environment in which PrEP was being rolled out was characterized by contrasting messages between the private donor-funded research organizations and the public health care providers who simply dismissed PrEP as being an ARV, without providing accurate information regarding ARVs being used in the form of PrEP to prevent HIV. This created a sense of uncertainty with regard to the legitimacy and efficacy of PrEP among FSWs.

PrEP prophylaxis as an ARV is being rolled out in an environment where HIV stigma is rife. HIV stigma originates from the association of HIV acquisition with socially unacceptable behavior or promiscuity; therefore, those contemplating or taking PrEP do not want to be associated with HIV medication, which in turn confers HIV-related stigma. PrEP is described as socially discrediting because PrEP is an ARV and by its association with HIV medication it is stigmatized (40). The stigma incurred by PrEP is attached to the persistent stigma toward people living with HIV, despite 30 years of HIV awareness campaigns and advocacy. Studies have shown that the stigma associated with HIV has not been sufficiently abated (41–45). Various research studies among other high-risk groups such as MSM, echo similar concerns regarding the fear of being labeled HIV positive because of the choice to take PrEP (40, 46, 47). As a result of societal disapproval, individuals who experience stigma tend to internalize the negative attitudes that they experience. This results in internalized stigma, characterized by feelings of shame, guilt, and worthlessness. Studies that have investigated internalized stigma among HIV-positive individuals, have reported that internalized stigma has prevented HIV-positive individuals from initiating ARV treatment because of internalized expectations of rejection, which may lead to withdrawal, isolation, secrecy, and non-disclosure of HIV status to partners (48, 49). Similarly, stigma-related challenges need to be anticipated and addressed for FSWs contemplating to take PrEP. This is particularly important for FSWs because they are most likely to experience intersecting stigma for engaging in sex work (50).

Another source of stigma was as a result of the targeted approach to PrEP. The argument made by FSWs in this study was that this targeted approach reinforced the notion that FSWs are the carriers of HIV. Furthermore, some participants in this study argued that this approach side-lined other women who may be at risk for HIV but are not identified as sex workers, such as women engaging in transactional sex. Most participants felt strongly about the need for the distribution of PrEP to be available to all people who are sexually active. They felt that this would facilitate more awareness and education around PrEP and, in turn, lessen the stigma. Similarly, there are studies that advocate for the normalization of PrEP through wider societal distribution (15, 51).

For effective PrEP uptake, it is important for FSWs to have an accurate perception of risk. The findings from this study showed that young girls selling sex, as well as new entry FSWs, have difficulty in navigating the high-risk exposure in sex work. The reasons provided were that young girls are unable to negotiate condom use with clients because of the power imbalances in relationships due to issues associated with hegemonic masculinity where men dictate the terms of engagement in sexual transactions (52, 53). Young women were also said to focus more on making money fueled by their desirability, seen as ‘fresh’ by men, as opposed to older sex workers. Another reason provided for ineffective risk assessment on the part of older new entry FSWs is that some FSWs are oblivious to the risky lifestyles they lead because they enter sex work gradually through transactional relationships that lack the boundaries of sex work, whereby condoms are expected and at times successfully negotiated. Various studies have shown that accurate risk perception plays an important role in effective HIV prevention (54–56). Research published from other PrEP trials such as the Pre-exposure Prophylaxis Trial for HIV Infection among African Women, found that FSWs who did not accurately assess their own exposure to risk, were less likely to prevent HIV (57).

The majority of FSWs in this study reported that they experienced interpersonal conflict because their HIV-positive friends see them as being in denial of their HIV status. This conflict is fueled by jealousy due to HIV acquisition being an expected by-product of engaging in sex work. Some participants recounted incidents of conflict which led to the breakdown of relationships with their intimate partners because they did not disclose that they were taking PrEP out of fear that the partner will think that they are HIV positive or sexually promiscuous. Some decided not to disclose because they felt ill-equipped to explain the mechanisms of PrEP being an ARV for HIV prevention. Moreover, the participants felt that the use of PrEP by one partner in the relationship has the potential to raise questions about trust and fidelity which may result in conflict (40). Results from a study among MSM showed that the reaction or support from partners, peer educators and family could either be a facilitator or barrier to PrEP use (58). In a multi-country study, 68% of the participants who formed part of a sample of young women and MSM, replied that they would definitely like for their partner to know that they are taking PrEP (59). In a study among female partners of migrant miners (60), the women felt that the reaction of their husbands toward them taking PrEP could either be a motivating or demotivating factor to uptake (60). Some women in that study felt that it was important to talk to their partners about PrEP and expressed a moral obligation to inform their partners that they were taking PrEP. Others felt that in some instances it might be necessary to broach the subject with their husbands, and judging by their reaction, they may decide to take PrEP without their partners' knowledge. Some male partners were adamant that if they found out that their wife was taking PrEP secretly, there would be consequences which could lead to divorce (60). All these examples demonstrate the impact that male partner influence has on the decision a woman makes regarding PrEP uptake.

According to the participants in this study, the messaging on PrEP is not clear because health care providers at public clinics and hospitals simply dismiss PrEP as ARV for people living with HIV, without providing a clear differentiation of the two regimens either as a source of prevention or treatment. This miscommunication has contributed to some FSWs either stopping to take PrEP or needing further convincing by peer educators from the research organizations that provide PrEP (47). Studies have shown that it is important to have effective follow-up systems to help PrEP users adhere and clarify any misconceptions that may arise. The peer education approach has been proven to be an effective way for disseminating knowledge and encouraging HIV prevention inclusive of PrEP among FSWs (61, 62). However, there is a need to couple peer education with other community-based structural and behavioral interventions as encouraged by the combination prevention framework (63).

A study that explored the acceptability of oral PrEP prior to implementation, provided insights from FSWs to ensure effective PrEP uptake, and the suggestion was that clear and accurate education and messaging are important (15). However, the results from this study have shown that this is not yet the case because of the confusion that exists among HIV-negative and HIV-positive FSWs in relation to what PrEP is and how PrEP works. Another issue reported in this study that cast doubt regarding PrEP efficacy, is the fact that it was available through research organizations implying that it is still in the development phase and it was being tested on FSWs. There have been various randomized control trials that have involved FSWs in microbicides (vaginal and anal) PrEP and those products did not become mainstream HIV prevention methods (64, 65). The participants in this study and other studies suggested that PrEP should be promoted to the broader community and be made available to others to reduce the stigma which resulted because of a lack of knowledge about PrEP (16). Evidence showed that the lack of awareness about PrEP is generally still high. A systematic review of studies from low- and middle-income countries found that of the 13 quantitative studies conducted in various parts of the world that reported on awareness of PrEP among MSM, only a total of 29.7% of MSM were aware of PrEP; however, the awareness did not necessarily translate into an understanding of PrEP and its clinical components (58). In a study conducted in India, a few of the participants confused PrEP with post-exposure prophylaxis. Evidently, there is a dearth of studies that reported on the statistical representation of PrEP awareness among FSWs, particularly in sub-Saharan Africa (66); therefore, more PrEP awareness and education coupled with behavioral interventions are needed to facilitate better uptake and possible retention.

## Conclusion

In conclusion, this paper has established the barriers to effective PrEP uptake and retention among FSWs. The barriers identified, were PrEP stigma which was one of the main hindrances to PrEP uptake. PrEP stigma occurred as a result of PrEP being associated with being HIV positive and thus with ARV treatment which is still highly stigmatized. The concern among PrEP users was that they did not want to be identified as HIV positive because of taking PrEP. The labeling of PrEP users as high risk was a compounding factor to PrEP stigma because it reinforced the notion of FSWs as drivers of HIV. These factors made it difficult for FSWs to open up to their intimate partners and friends about PrEP because of its association with HIV. This also led to conflict among FSWs with their HIV-positive friends who failed to understand that ARV treatment can be used for prevention purposes in the form of PrEP. The root of this conflict was intensified by the prevalent belief among FSWs that all FSWs are HIV positive because they share clients and, therefore, some felt that it was not possible to be on ARVs and be HIV negative. Another barrier to early prevention was inaccurate assessment of risk, particularly among adolescents entering the sex trade, as well as older FSWs who are oblivious to the risks incurred in the trade. These groups were unlikely to take up PrEP for early HIV prevention. The participants felt that it was important for the South African government to avoid the targeted approach and provide PrEP at a larger scale to people of all ages who are sexually active and HIV negative, as this will promote widespread PrEP education to curb the PrEP-related stigma. There was also a call for a synchronized approach to PrEP provision among research organizations and public health facilities who provide PrEP. As a recommendation, public health care providers need to be trained on the uses of ARVs, both as a treatment for HIV as well as a prevention option in the form of PrEP. FSWs need to be assisted on how they can explain their use of PrEP to their intimate partners, and more PrEP education is needed among FSWs to promote risk awareness and early prevention, as well as to correct the misconceptions about HIV prevalence in this group. Programs need to encourage both HIV-negative and HIV-positive FSWs to support one another in daily pill intake to avoid conflict and misunderstanding, as well as to create a cohesive HIV prevention environment for both groups.

### Limitations

This is a qualitative study and findings cannot be generalized to the broader sex work population in South Africa. The use of snowball sampling to recruit participants means that we missed perspectives from FSWs from other race groups and networks.

## Data Availability Statement

The raw data supporting the conclusions of this article will be made available by the authors, without undue reservation.

## Ethics Statement

The studies involving human participants were reviewed and approved by Humanities and Social Sciences Research Ethics Committee. The patients/participants provided their written informed consent to participate in this study.

## Author Contributions

NM conceptualized the study, collected and analyzed the data and wrote the manuscript. AM-W reviewed the manuscript and provided critical feedback which helped shape the development of the manuscript. YS also reviewed the manuscript and provided feedback assisting in the final preparations of the manuscript for submission. All authors contributed to the manuscript revision, read and approved the submitted version.

## Funding

This work is based on the research supported wholly/in part by the National Research Foundation of South Africa (Grant Numbers: 129667).

## Conflict of Interest

The authors declare that the research was conducted in the absence of any commercial or financial relationships that could be construed as a potential conflict of interest.

## Publisher's Note

All claims expressed in this article are solely those of the authors and do not necessarily represent those of their affiliated organizations, or those of the publisher, the editors and the reviewers. Any product that may be evaluated in this article, or claim that may be made by its manufacturer, is not guaranteed or endorsed by the publisher.

## Abbreviations

ARVs: antiretrovirals
FSWs: female sex workers
HIV: human immunodeficiency virus
MSM: men who have sex with men
PrEP: pre-exposure prophylaxis
TB: tuberculosis.
